# Prevalence of Cobalt in the Environment and Its Role in Biological Processes

**DOI:** 10.3390/biology12101335

**Published:** 2023-10-16

**Authors:** Giuseppe Genchi, Graziantonio Lauria, Alessia Catalano, Alessia Carocci, Maria Stefania Sinicropi

**Affiliations:** 1 Dipartimento di Farmacia e Scienze della Salute e della Nutrizione, Università della Calabria, Arcavacata di Rende, 87036 Cosenza, Italy; giuseppe.genchi@unical.it (G.G.); glauria@unical.it (G.L.); s.sinicropi@unical.it (M.S.S.); 2 Dipartimento di Farmacia-Scienze del Farmaco, Università degli Studi di Bari “A. Moro”, 70125 Bari, Italy; alessia.carocci@uniba.it

**Keywords:** cobalt, vitamin B_12_, cobalt-dependent enzymes, toxicology, phytoremediation, cobalt nanoparticles

## Abstract

**Simple Summary:**

Cobalt (Co) is an essential element with ubiquitous dietary exposure and possible incremental exposure due to dietary supplements, occupations, and medical devices. The main function of Co in humans is based on its role in vitamin B_12_ (cobalamin). This review provides an extended overview of Co in relation to its effects on health and biological processes.

**Abstract:**

Cobalt (Co) is an essential trace element for humans and other animals, but high doses can be harmful to human health. It is present in some foods such as green vegetables, various spices, meat, milk products, seafood, and eggs, and in drinking water. Co is necessary for the metabolism of human beings and animals due to its key role in the formation of vitamin B_12_, also known as cobalamin, the biological reservoir of Co. In high concentrations, Co may cause some health issues such as vomiting, nausea, diarrhea, bleeding, low blood pressure, heart diseases, thyroid damage, hair loss, bone defects, and the inhibition of some enzyme activities. Conversely, Co deficiency can lead to anorexia, chronic swelling, and detrimental anemia. Co nanoparticles have different and various biomedical applications thanks to their antioxidant, antimicrobial, anticancer, and antidiabetic properties. In addition, Co and cobalt oxide nanoparticles can be used in lithium-ion batteries, as a catalyst, a carrier for targeted drug delivery, a gas sensor, an electronic thin film, and in energy storage. Accumulation of Co in agriculture and humans, due to natural and anthropogenic factors, represents a global problem affecting water quality and human and animal health. Besides the common chelating agents used for Co intoxication, phytoremediation is an interesting environmental technology for cleaning up soil contaminated with Co. The occurrence of Co in the environment is discussed and its involvement in biological processes is underlined. Toxicological aspects related to Co are also examined in this review.

## 1. Introduction

Cobalt (Co), discovered in 1735 by G. Brandt, is a transition metal that is between iron and nickel in the Mendeleev periodic table [1]. Co occurs in the ground state of biological systems in oxidation states of +2 and +3. Co is an essential trace element for prokaryotes, animals, and humans [2]. It is a pivotal component of vitamin B_12_ (cobalamin), takes part in metabolism by stimulating hematopoiesis, enhances the immune response, and demonstrates antibacterial activity. Within vitamin B_12_, Co may exist in the +3 oxidation state in an octahedral geometry. In the plane, Co is coordinated to four nitrogen atoms of a corrin ring. As a lower ligand, it is linked to 5′,6′-dimethylbenzimidazole, and, as an upper ligand, to hydroxide, methyl, cyanide, or 5′-deoxyadenosyl.

The diverse forms of cobalamin are needed for DNA synthesis, for the production of red blood cells and fatty acids, for the synthesis of amino acids, and for normal brain and nerve functions. Methylcobalamin and adenosylcobalamin are the metabolically active derivatives of vitamin B_12_, while cyanocobalamin, the most stable form of vitamin B_12_, is inert and is not utilized as a cofactor [3], even though it is often present in dietary supplements [4]. After its absorption in the gastrointestinal tract, cyanocobalamin is partially converted to biologically active compounds [5]. Mammals are not able to synthesize vitamin B_12_, unlike some bacteria and archaea [6]. Co is a cofactor of different enzymes and is a component of several proteins in prokaryotes and animals [7]. Nitrogen fixation bacteria require Co for fixing nitrogen, N_2_, in the atmosphere into ammonia, NH_3_, to furnish the nitrogen macronutrients to plants [2,8]. Vitamin B_12_ belongs to the “corrinoids” group, which comprises compounds that contain a corrin macrocycle. The term “Vitamin B_12_” is usually restricted to cyanocobalamin, which is the most chemically stable and a non-natural form of cobalamin [9], being an artifact of its isolation from microbial fermentation. Several activities have been reported for corrinoids, such as being catalysts of biologically important organic reactions, as well as their involvement in photochemistry and photobiology processes [10]. However, some corrinoids, such as those contained in edible algae and edible mushrooms are inactive [11]. In contrast to other transition metals, Co is generally used as a cofactor in the corrinoid form. Only eight non-corrin cobalt-containing enzymes are known so far, of which, nitrile hydratase and methionine aminopeptidase are the best studied. Cobalt is best known for its central role in alkylcorrinoid cofactors, where the unique properties of the cobalt–carbon bond are exploited to catalyze chemically challenging biotransformations [12]. Co^2+^ and Co^3+^ ions are essential for plants and animals as trace elements. Co may be introduced in the organism by diet, respiration, and contact with soil and water containing this element. Co compounds are used for painting in ceramics and glass. Moreover, Co is present in superalloys, other alloys, heavy metals (cement carbides), magnets, wear-resistant materials, recording materials, batteries, tires, paint dryers, soaps, and catalysts [13]. Recently, Co–chromium balloon-expandable vascular stents have been used in congenital heart disease interventions [14]. 

Finally, in recent years, there has been a growing interest in nanoparticle systems due to their unique properties which make them applicable in different fields from microelectronics to medicine [15,16,17]. Various chemical and physical processes are used to produce metallic nanoparticles. In addition, these nanoparticles can be modified for specific tasks as a consequence of post-synthetic modification reactions. Co nanoparticles and cobalt oxide nanoparticles can be used in different fields thanks to their physico-chemical properties and small size (10–500 nm in diameter). Co nanoparticles have been used for electrochemical applications, the production of batteries, gas sensors, catalysts, and a variety of biomedical applications [18,19]. In addition, in some infectious diseases Co nanoparticles can be used as antibacterial, antifungal, and antiparasitic agents [20]. Moreover, Co nanoparticles can be utilized to carry drugs or antigens encapsulated for targeted cancer therapy [21,22]. Finally, Co nanoparticles are used as a catalyst in the hydrolysis of natrium borane to produce hydrogen to decompose various dyes [23]. Although this is an essential metal, high concentrations of Co pose a risk to human health [24] by decreasing pulmonary function, leading to eye disorders, thyroid damage, and neurological and heart diseases [25]. Thus, it is often described as a heavy metal with related toxicity [26]. Co is released into the environment by volcanic eruptions, fossil fuel combustion, surface runoff, copper, iron, and nickel smelting and refining, alloy manufacturing, battery production, and agricultural phosphate fertilizers. Detoxification from Co may be obtained by the use of chelating agents. Phytoremediation treatments are a cost-effective and environmentally friendly technology for the reduction of Co contaminations. Phytostabilization, phytoextraction, and rhizofiltration are some other techniques utilized to reduce Co in the field. This review provides information, based on highlighted literature studies, regarding the prevalence of Co in the environment and its role in biological processes and biomedical applications. New technologies for reducing Co contamination in the environment are also described.

## 2. Chemical and Physical Properties of Cobalt

Co is a transition element with the atomic number 27, atomic weight 58.93, and density of 8.90 g/cm^3^. It is one of the three ferromagnetic metals, along with iron and nickel, of Group 9 (VIII) of the periodic table [27,28]. This metal was isolated by Swedish researcher George Brandt in 1735 [1]. Co dissolves in dilute acid and interacts with carbon, sulfur, and phosphorous. At high temperatures, it forms cobaltous oxide, CoO, by reacting with oxygen and water vapor. The oxidation states of Co are −3, −1, 0, +1, +2, and +3. Among them, the most common are +2 and +3. Metallic Co occurs in two allotropic hexagonal and cubic forms [29]. Co^2+^ is more stable than Co^3+^; the latter is a powerful oxidizing agent [30]. The standard reduction potential of free Co(III) ions is +1.842 V in aqueous solution [31]. Co comprises 0.0029% of the Earth’s crust; free Co (Co^0^) is not found on Earth because it easily reacts with atmospheric oxygen and chlorine present in the water of rivers, lakes, and oceans. Co is a steel-gray, shiny, brittle metal that resembles iron and nickel, and is ductile and malleable. Co and nickel are components of meteoritic iron, even if in these meteorites Co is much less abundant than nickel, as well as in the sun and stellar atmospheres [32]. Co is produced usually as a byproduct of cuprum, nickel, iron, and silver mining and it is also present in deep-sea nodules. The Democratic Republic of the Congo, China, Canada, and Russia are the world’s leading producers of mined cobalt. At present, the Democratic Republic of the Congo produces about 70% of the world’s Co [33,34]. ^59^Co is the only stable Co isotope present naturally on Earth. Twenty-two radioisotopes of Co have been characterized; among these ^60^Co is radioactive with a half-life of 5.274 years and decays to nonradioactive nickel (^60^Ni) [35].

## 3. Role of Cobalt in Physiological Processes

### 3.1. Vitamin B_12_ (Cobalamin): The Vitamin with Co

Vitamin B_12_ is a water-soluble vitamin essential for the synthesis of DNA, and for the mitochondrial methylmalonyl-CoA mutase, which enters the β-oxidation reaction of odd chain fatty acids. It was identified in 1948 [36], when two research groups from pharmaceutical companies (Folkers at Merck, Sharp & Dohme and Smith at Glaxo) isolporphated, almost at the same time, a cobalt compound from animal livers that was able to cure pernicious anemia, a condition characterized by immature and abnormal red blood cells [37]. The molecular formula is C_63_H_88_CoN_14_O_14_P and the molecular weight 1355.4 g/mol. Only 4.34% in the weight of the molecule is represented by Co. 

Vitamin B_12_ is the only vitamin with which a metal ion is associated. It is a cofactor for the highly conserved enzymes methionine synthase and methylmalonyl-CoA mutase, which function in amino acid synthesis and fatty- and amino acid breakdown, respectively, in both bacteria and mammals; therefore, it plays a key role in homeostatic functions. Indeed, in humans, cobalamin deficiency can lead to decreased activity of methionine synthase and methylmalonyl-CoA mutase, resulting in megaloblastic anemia [38]. Vitamin B_12_ also plays an essential role as a coenzyme in many biochemical processes that maintain or restore the health of the nervous system [39]. It is involved in the synthesis of myelin. The myelin sheath surrounds the axons of several nerves and behaves as an electrical insulation, thus facilitating fast conduction velocity. The contribution of vitamin B_12_ to myelin formation and remyelination supports the regeneration of nerves after an injury [40].

The biosynthesis of vitamin B_12_ is carried out by bacteria and archaea. About 30 enzyme-mediated steps are involved in converting aminolevulinic acid via uroporphyrinogen III and adenosylcobyric acid to cobalamin. Uroporphyrinogen III is the first macrocyclic intermediate to heme and chlorophyll. Biochemically, vitamin B_12_ consists of four pyrrolic nitrogens coordinating a central atom of cobalt in the Co^3+^ oxidation state (corrin ring). The corrin pyrrolic groups are equipped with eight methyl groups, acetamide and propionamide groups, one of which is bound to dimethylbenzimidazole. Two routes for the synthesis of cobalamin are known, namely anaerobic (cobalt early) and aerobic (cobalt late), which differ in the timing of cobalt insertion [41]. The feature that distinguishes these two biosynthetic routes is whether the cobalt is incorporated early in anaerobic organisms or late in aerobic organisms in the presence of oxygen, which helps the contraction process and the cobalt insertion after all the methylations have taken place [42].

Vitamin B_12_ is involved in homocysteine metabolism, nerve metabolism, fatty acid and nucleic acid synthesis, energy production, and cell maturation processes, and also supports the maintenance of the undamaged gastrointestinal mucosa. Moreover, vitamin B_12_ levels impair the amount of reduced glutathione with antioxidant activity in the erythrocytes and in the liver; thus, the lower availability of reduced glutathione in cobalamin deficiency may expose cells to increased oxidative stress [43]. 

There is evidence that vitamin B_12_ plays a crucial role in the healthy balance of the immune system. Inadequate levels of vitamin B_12_ and folic acid (necessary for DNA synthesis) can dramatically alter immune responses by affecting the production of nucleic acid and protein synthesis, inhibiting the activity of immune cells, and interfering with metabolic processes, such as methylation and serine, glycine, and purine cycles. Inefficient methylation can lead to hyperhomocysteinemia which causes systemic and vascular inflammation, contributing to the pathogenesis of many other diseases [44]. Several studies have shown that high levels of homocysteine and low levels of vitamin B_12_ and folate are associated with an increased risk of developing Alzheimer’s disease [45,46]. Recently, the role of vitamin B_12_ in the treatment of Alzheimer’s disease [47,48] and viral infections, including COVID-19 [49,50], have been highlighted. Moreover, a deficiency in vitamin B_12_ in older adults has been suggested to be implicated in ischemic stroke [51].

**Figure 1:**
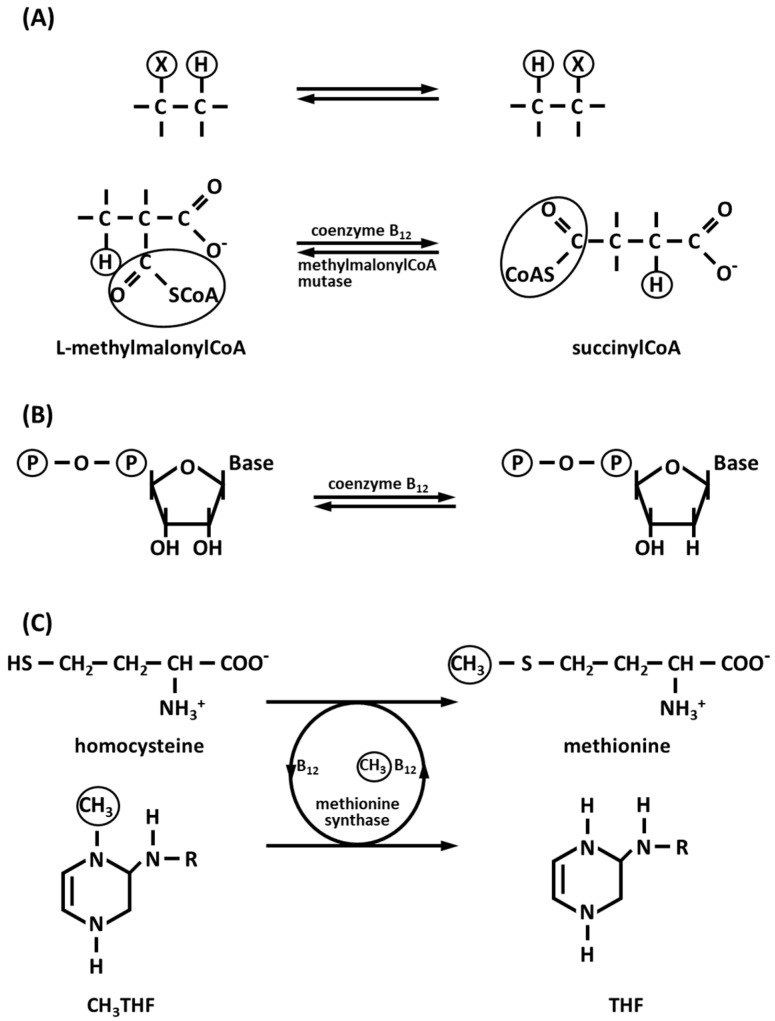
Reactions catalyzed in the presence of coenzyme B_12_. (**A**) Intramolecular rearrangement (in the intramolecular rearrangement, catalyzed by methylmalonyl-CoA mutase, the group -CO-SCoA at C-2 of L-methylmalonylCoA exchanges with a hydrogen atom at C-3 of the same substrate in the presence of coenzyme B_12_ to yield succinylCoA. This coenzyme generally catalyzes an exchange of an alkyl group (X) with an adjacent hydrogen atom); (**B**) Ribonucleotide reduction (coenzyme B_12_-dependent ribonucleotide reductases catalyze the reduction of ribonucleotides to deoxiribonucleotides and are essential for de novo synthesis and repair of DNA); (**C**) Methyl group transfer; CH_3_THF, methyltetrahydrofolate: THF, tetrahydrofolate (the enzyme methionine synthase catalyzes the transfer of a methyl group from 5-methyltetrahydrofolate to homocysteine to generate methionine in the presence of the cofactor cobalamin, that serves as both acceptor and donor of methyl group).

Vitamin B_12_ is converted in the body into two coenzymes B_12_: methylcobalamin and 5′-deoxyadenosylcobalamin. These two coenzymes are the metabolically active derivatives of vitamin B_12_ and are about 75–90% and 10–25% of the total vitamin B_12_ pool. English crystallographic chemist Dorothy Crowfoot Hodgkin and collaborators (1956) [52] determined the complex three-dimensional structure of this vitamin from a crystallographic X-ray diffraction study. The complex structure of this coenzyme consists of a corrin ring system, to which a Co ion (as Co^3+^) is coordinated in the center. The corrin ring with four pyrrole groups is similar to the porphyrin ring system of heme and heme proteins. The Co is coordinated to the four planar pyrrole nitrogens. The difference between a corrin and a porphyrin is the direct linkage between the A and D rings in the former. This results in a smaller central cavity which probably has implications for the chemistry of Co [10]. One of the axial Co ligands is a nitrogen of a dimethylbenzimidazole ribonucleotide group, while the other axial ligand may be –CH_3_, –OH, –CN, or the 5′-carbon of 5′-deoxyadenosine. Intramolecular rearrangement (Figure 1A) and reduction of ribonucleotides to deoxyribonucleotides (Figure 1B) are catalyzed in the presence of 5′-deoxyadenosylcobalamin, while methyl group transfer is mediated in the presence of the cytoplasmic enzyme methionine synthase and methylcobalamin in the conversion of homocysteine to methionine (Figure 1C) [53]. 

The conversion of homocysteine to methionine prevents the accumulation of homocysteine in serum, limiting vascular diseases, neurological abnormalities, and brain atrophy. Furthermore, the production of the essential methionine allows the synthesis of proteins, DNA and RNA, due to the methylation reaction [54]. Coenzyme B_12_ and the mitochondrial enzyme methylmalonyl-CoA mutase enter the process of β-oxidation of odd-chain fatty acids [53]. These fatty acids are common in lipids of plants and marine organisms, that so enter the human diet. Fatty acids with an odd number of carbon atoms are oxidized following the same metabolic pathway of fatty acids with an even number of carbon atoms, releasing at each cycle acetylCoA, which is oxidized in the Krebs citric acid cycle. In the last cycle, a fatty acylCoA of five carbon atoms is formed, which is cleaved producing acetylCoA and propionylCoA. AcetylCoA can be oxidized in the citric acid cycle, while propionylCoA enters a different enzymatic pathway involving three more enzymes. PropionylCoA carboxylase in the presence of CO_2_, the cofactor biotin, and ATP, forms D-methylmalonylCoA, which is enzymatically epimerized to L-methylmalonylCoA by methylmalonylCoA epimerase. The third reaction, catalyzed by methylmalonyl-CoA mutase vitamin B_12_-dependent is unusual [55]; in fact, it involves an intramolecular rearrangement, in which an exchange occurs between a hydrogen atom and its neighbor carbonyl group to form succinylCoA, starting from L-methylmalonylCoA (Figure 1A). SuccinylCoA enters the citric acid cycle. Vitamin B_12_ is present in dietary food such as meat, especially liver and kidney, even if mammals are not able to synthesize this vitamin. Vitamin B_12_ is synthesized only by microorganisms present in the intestinal flora. 

The presence of vitamin B_12_ in animal products is due to microbial activity and these aliments, taken up with the diet, are the only natural source for humans. Vegans can be at risk of dietary deficiency of vitamin B_12_ because their diet, based only on plant foods (vegetables, grains, nuts, and fruits), does not contain this vitamin. Vegans do not eat foods that come from animals, including dairy products and eggs. Vegan diets have to include fortified foods and supplements containing cyanocobalamin (the synthetic form of vitamin B_12_), vitamin D, selenium, iodine, calcium, iron, and nutritional yeast. Vitamin B_12_ is synthesized by some intestine-resident bacteria and archaea, such as *Bacillus megaterium*, *Escherichia coli*, *Fervidobacterium nodosum*, *Nitrosopumilus maritimus*, *Propionibacterium shermanii*, *Pseudomonas denitrificans*, *Salmonella typhimurium*, *Termosipho africanus*, and *Thermotoga* sp. RQ2 [56]. 

Vitamin B_12_ is bound to proteins in food; in the stomach, it is released by gastric acid breakdown and then bound to the protein intrinsic factor. The nutritional requirement for this vitamin is very low, about 3–5 µg per day. Oral ingestion is the entry point into vitamin B_12_ metabolism, followed by its transport and uptake via three binding proteins, namely, haptocorrin, intrinsic factor, and transcobalamin (TC), and their respective membrane receptors. The three proteins act sequentially, at specific points of the food-to-tissue delivery route. Inherited mutations of genes encoding intrinsic factor (*CBLIF*), its receptor (*CUBN, AMN*), and transcobalamin (*TCN2*) are causative of disease, while the clinical relevance is uncertain for mutations of genes encoding the transcobalamin receptor (*CD320*) as well as haptocorrin (*TCN1*), which has no dedicated receptor. Haptocorrin is secreted by salivary glands, and first binds the vitamin in the stomach, to protect it from gastric acid hydrolysis. This complex travels through the upper gastrointestinal tract to the duodenum, where haptocorrin is degraded by pancreatic proteases, releasing vitamin B_12_. Then, the free vitamin in the duodenum is bound by intrinsic factor [57]. This complex continues through the small intestine until it is internalized by enterocytes of the distal ileum through recognition by a receptor consisting of cubilin and amnionless [58]. After enterocyte internalization, vitamin B_12_ is transported through the cell and exported into the blood. Approximately 80–90% of plasma vitamin B_12_ is bound by haptocorrin. The remaining 10–20% is bound by TC. Interaction of the complex trancolbalamin–vitamin B_12_ with the receptor megalin mediates renal reabsorption, while recognition by the TC receptor is responsible for uptake in liver and other tissues.

Vitamin B_12_ deficiency results in serious anemia, a condition in which there is an inadequate amount of hemoglobin to carry oxygen to tissues. Megaloblastic anemia is caused by a deficiency of vitamin B_12_ and folate and is characterized by immature, large, and abnormal red blood cells [59]. Pernicious anemia does not result from a dietary deficiency of vitamin B_12_ but from the absence of the intrinsic factor [60]. Serum vitamin B_12_ concentration declines during pregnancy, particularly in multiple pregnancies. The recommended daily allowance for B_12_ increases during pregnancy from 2.4 to 6.0 μg/day. It is noteworthy that people at high risk of development of vitamin B_12_ deficiency are vegetarians and vegans, since this vitamin is not available from plant sources [61]. Often, a vitamin B_12_-deficient mother is associated with B_12_ deficiency in a newborn infant [62,63]. Moreover, metformin-treated type 2 diabetes mellitus patients are at higher risk of vitamin B_12_ deficiency and neuropathy. However, there is still no guideline suggesting vitamin B_12_ supplementation for this population [64].

### 3.2. Cobalt Corrinoids

Cobalt corrinoids are derivatives of vitamin B_12_ [65,66,67], which often catalyze organic reactions [68] and are involved in photochemistry and photobiology reactions [69,70]. Moreover, the function of vitamin B_12_ and corrinoids as riboswitches is known [71]. However, in various plant-based foods, substantial amounts of corrinoids that are inactive, such as pseudovitamin B_12_, adenyl cobamide, factor IV, pseudocobalamin, 5-methoxybenzimidazolyl cobamide, 2-methylmercaptoadenyl cobamide, and cobalamin[c-lactone], may be found [11,72,73].

Corrinoid metabolism in bacteria and archaea is regulated by riboswitches, RNA regulatory elements, which are noncoding RNA, that bind their ligands like vitamins, nucleotides, and amino acids with high affinity. Cobalamin riboswitches are prevalent throughout the bacterial kingdom and regulate the gene products associated with corrinoid biosynthesis, porphyrin, and B_12_-independent ribonucleotide reductase. Cobalamin riboswitches are divided into two classes, namely, aquacobalamin and adenosylcobalamin riboswitches, depending on their metabolite [74].

The energy of sunlight is converted into chemical energy by photosynthetic bacteria, algae, and plants; and the ability to respond to light is vital for living organisms. Living organisms respond to light using photoreceptors, which rely upon retinal, flavins, linear tetrapyrroles, carotenoids, deoxyadenosylcobalamin, and methylcobalamin: these unsaturated molecules have a conjugated π system that allows light absorption. As a result of light absorption, cobalamins exhibit complex photochemistry, which is modulated by upper and lower axial ligands. Near-UV and visible light (wavelengths below 530 nm) cleave the Co–C bond of both methylcobalamin and deoxyadenosylcobalamin at a time of about 10–100 picoseconds. Under aerobic conditions, in the case of methylcobalamin, the highly reactive methyl radical forms formaldehyde and smaller amounts of methanol, formic acid, and carbon dioxide, while under anaerobic conditions, it forms formaldehyde, methane, ethane, and smaller amounts of methanol and formic acid. After photolysis of deoxyadenosylcobalamin, the formed deoxyadenosyl radical reacts immediately with oxygen to form peroxyadenosine, which, in turn, decomposes to adenosine-aldehyde with minor amounts of adenosine and adenine. In the absence of oxygen, deoxyadenosyl radical reacts with adenine, forming deoxy-5′,8-cycloadenosine [74].

### 3.3. Co-Dependent Enzymes

Co is an essential trace element in both eukaryotes and prokaryotes, but it is present less frequently in metalloproteins compared to other transition metals. At present, eight noncorrin Co-dependent enzymes have been isolated and characterized: methylmalonyl-CoA carboxytransferase, prolidase, methionine aminopeptidase, nitrile hydratase, glucose isomerase, aldehyde decarbonylase, lysine-2,3-aminomutase, and bromoperoxidase.

#### 3.3.1. Methylmalonyl-CoA Carboxytransferase 

**Figure 2:**
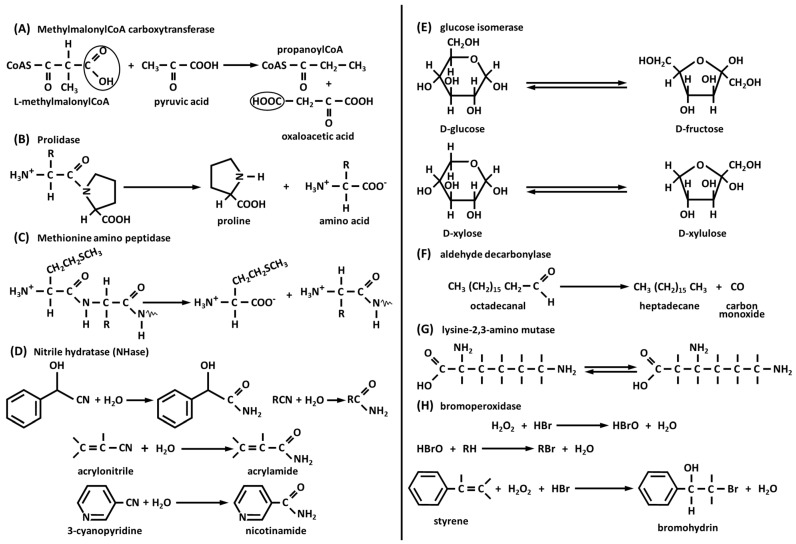
Reactions catalyzed by Co-dependent enzymes: (**A**) Methylmalonyl-CoA carboxytransferase (the methylmalonyl-CoAcarboxytranferase catalyzes the transfer of carboxyl group to pyruvic acid from methylmalonylCoA to produce propanoylCoA and oxaloacetic acid); (**B**) Prolidase (R = Met, Val, Phe, Ala, Leu) (the enzyme prolidase hydrolyzes dipeptides formed from Pro (C-terminal) and Ala, Leu, Met, Phe, or Val (N-terminal)); (**C**) methionine aminopeptidase (R = Ala, Gly, Ser, Cys, Pro, Val) (methionine aminopeptidase removes from proteins the N-terminal methionine, when the penultimate aminoacid is Ala, Cys, Gly, Pro, Ser, or Val); (**D**) Nitrile hydratase, glucose isomerase, aldehyde decarbonylase, lysine-2,3-aminomutase, and bromoperoxidase (the Co^3+^ and Fe^2+^-dependent nitrile hydratase catalyzes the addition of water to nitrile moieties. The industrial production of acrylamide starting from acrylonitrile utilizes this enzyme); (**E**) Glucose isomerase (glucose isomerase catalyzes the reversible reaction of aldoses to ketoses. Important commercial reactions are the isomerization of D-glucose to D-fructose and D-xylose to D-xylulose); (**F**) Aldehyde decarbonylase (the synthesis of long chain alkane waxes in animals, plants, and microorganisms involves the presence of the enzyme aldehyde decarbonylase that removes one carbon monoxide from long chain aldehydes); (**G**) Lysine-2,3-aminomutase (lysine-2,3-aminomutase catalyzes the interconversion reaction of 2-lysine to 3-lysine that is the precursor of the synthesis of the antibiotics vimycin, nourseothricin, and streptothricin); (**H**) Bromoperoxidase (R = organic molecules) (this bromoperoxidase enzyme, in the presence of hydrobromic acid and hydrogen peroxide, catalyzes the bromination of styrene and indene, producing the corresponding bromohydrins).

This is a 1.2 million Da molecular weight multienzyme complex of 30 polypeptides from *Propioniibacterium shermanii*. This enzyme transfers a carboxyl group from methylmalonyl-CoA to pyruvate (Figure 2A), yielding propanoyl-CoA and oxaloacetate. This complex consists of a dimer of β8α8 barrels with Co^2+^ octahedrally coordinated by a carbamylated lysine, a water molecule, one Asp, and two His [75]. The 12S subunit transfers a carboxylate group from methylmalonylCoA to the biotin of the 1.3S subunit forming carboxylate 1.3S biotin and propanoyl-CoA.

#### 3.3.2. Prolidase 

This hydrolyzes only dipeptides with proline at the C-terminal and Met, Val, Phe, Ala, or Leu at the N-terminal (Figure 2B) [76]. The enzymatic activity of this enzyme releases proline and hydroxyproline during the breakdown of collagen, which is the most abundant structural protein in the human body. Prolidase is an important enzyme required in several pathological processes such as inflammation, angiogenesis, cell proliferation, and carcinogenesis [77]. This enzyme is produced during collagen turnover, and the pool of proline is reutilized during collagen synthesis. Its enzymatic activity requires the presence of a second Co^2+^ ion essential for catalysis. This second Co^2+^ can be replaced by Mn^2+^ with a loss of 25% of its catalytic activity. Protein synthesis in eukaryotes is initiated with methionine and in prokaryotes with formylmethionine. The mature proteins are subjected to N-terminal modification reactions, occurring post-translationally. Removal of N-terminal methionine in eukaryotes is catalyzed by methionine aminopeptidase; in bacteria, the N-formyl residue of N-formylmethionine is first removed by a deformylase, and then N-methionine is removed by methionine aminopeptidase. Thanks to its important role, this enzyme is present in both eukaryotes (human and porcine) [78] and prokaryotes (*Escherichia coli*, *Salmonella typhimurium*, and *Bacillus subtilis*) [79]. 

#### 3.3.3. Methionine Aminopeptidase 

This removes, preferentially, the N-terminal methionine from proteins, especially when the penultimate residues are small amino acids like Ala, Cys, Gly, Pro, Ser, or Val (Figure 2C). *Escherichia coli* enzyme is a monomeric protein and is active with two Co^2+^; the catalytic high-affinity site contains His 171 residue, two Glu (204 and 235), and two Asp (97 and 108) [79]. 

#### 3.3.4. Nitrile Hydratase (NHase) 

These are Co^3+^- and Fe^3+^-dependent metalloenzymes that catalyze the addition of water to nitrile moieties (Figure 2D). Nitrile hydratase has been utilized for the industrial production of acrylamide from acrylonitrile [80]. Also, nicotinamide, for use in animal feed, is industrially produced by hydrolysis of 3-cyanopyridine [81]. Nitrile hydratases are composed of α and β subunits; α subunit provides three Cys residues and two mainchain amide nitrogens, while the second and seventh residues are Thr and Tyr, and the sixth position is occupied by water or hydroxide. The two Cys residues coordinated to the Co are oxidized to Cys-sulfinic acid (Cys-SO_2_H) and Cys-sulfenic acid (Cys-SOH), which are important to enzyme function. *Rhodococcus rhodochorous* J1 produces two kinds of enzymes, one of 520 kDa molecular weight and the other one of 130 kDa. Both nitrile hydratases are composed of α and β subunits; the largest enzyme has ten of each subunit and the smallest has two of each. The addition of nickel or iron to the growth medium leads to the formation of an enzyme without catalytic activity.

#### 3.3.5. Glucose Isomerase 

This is also known as xylose isomerase and catalyzes the reversible isomerization of D-glucose to D-fructose and D-xylose to D-xylulose (Figure 2E). Glucose isomerase is present in bacteria, fungi, and plants, interconverting aldoses to ketoses. The isomerization reaction of glucose to fructose is of commercial and industrial importance for the production of high-fructose corn syrup [82] and bioethanol [83]. Fructose corn syrup is slightly sweeter than sucrose and is utilized by diabetics. Immobilization of this enzyme in column reactors provides an efficient system for its easy recovery and reuse, lowering the cost of its use at milder conditions of pH (7.5–8.0) and temperature (55–60 °C) in the presence of Mg^2+^ as a stabilizer. The Co^2+^ ion is essential for the production of this enzyme by *Streptomyces albus*. This ion can be removed by EDTA, resulting in an activity loss. Glucose isomerase can catalyze the isomerization of xylose to xylulose produced from hemicellulose, which can be fermented, producing ethanol in the presence of yeasts such as *Saccharomyces cerevisiae*, *Saccharomyces pastorianus,* and *Schizosaccaromyces pombe*.

#### 3.3.6. Aldehyde Decarbonylase

Long-chain alkane waxes are synthesized by animals, plants, and microorganisms. Plants (leaves and stems) secrete very long chain alkane waxes of about 30 carbon atoms and, together with wax esters formed from aliphatic alcohols and carboxylic acids, serve as a waterproof barrier to prevent desiccation. A wide variety of branched and unsaturated hydrocarbons are synthesized by larvae of insects to prevent desiccation. The synthesis of long-chain hydrocarbons [84,85] in animals, plants, and microorganisms involves decarbonylation of aldehydes to alkanes by loss of carbon monoxide (CO) (Figure 2F). The *aldehyde decarbonylase* in *Botryococcus braunii* consists of α (66 kDa) and β (55 kDa) subunits and contains one cobalt–porphyrin per α and β pair of subunits. In plants and green algae, this decarbonylase enzyme appeared to be membrane-associated with 66 kDa integral protein. Experiments on crude microsomal preparations of pea leaves (*Pisum sativum*) and green algae (*B. braunii*) demonstrated that these extracts were capable of converting octadecanal to heptadecane (Figure 2F) [84]. 

#### 3.3.7. Lysine-2,3-Aminomutase

This enzyme catalyzes the interconversion reaction of 2-lysine and 3-lysine (Figure 2G) [86]. This interconversion reaction might appear like the adenosylcobalamide-dependent catalyzed reaction; however, lysine-2,3-aminomutase is activated by S-adenosylmethionine (SAM) but not by adenosylcobalamin. Lysine-2,3-aminomutase, to develop its maximal activity, must contain Co^2+^, [Fe]clusters, and pyridoxal 5’-phosphate. In *Streptomyces* spp., 3-lysine is the precursor of the biosynthesis of vimycin, nourseothricin, and streptothricin [87]. 

#### 3.3.8. Bromoperoxidase

This enzyme catalyzes the reaction shown in Figure 2H. Itoh et al. (1994) [88] have purified to homogeneity bromoperoxidase enzyme from the *Pseudomonas putida* IF-3 strain (68 kDa molecular weight), which produces the antibiotic pyrrolnitrin. The optimum pH for the enzyme activity is about 4.2, and it is stable in the pH range between 4 and 11. This enzyme contains a Co ion, but it does not contain prosthetic groups like flavin and cobalamin. The activity of this enzyme is activated by about 300% in the presence of Co^2+^. The gene encoding Co^2+^-activated bromoperoxidase-esterase was cloned from DNA of the *P. putida* IF-3 strain and expressed in *E. coli* [89]. To develop its activity, this enzyme requires the presence of acetic acid, propionic acid, butyric acid, H_2_O_2_, Br^−^, and Co^2+^. This enzyme catalyzed the bromination reaction of styrene (Figure 2H) and indene, producing the corresponding bromohydrins.

## 4. Applications of Cobalt in Medicine

### 4.1. Cobalt in Prostheses

Co is used for prosthetic applications, both for hip and knee replacements and in dentistry. The metal alloy Co-Cr used in dentistry is composed of at least 60% by weight Co and 30% Cr, and other metals such as molybdenum (Mo) in smaller quantities. Co improves mechanical characteristics such as rigidity, and chromium increases corrosion resistance and biocompatibility because the release of metal ions in the oral cavity can cause hypersensitivity or allergy [90,91]. Co-Cr alloys are almost half the density of gold-based alloys, making dental restorations and removable partial denture frameworks significantly lighter [92]. Furthermore, they have been used as alternatives to nickel–chromium (Ni-Cr) alloys to avoid the risk of allergic reactions and toxicity related to Ni release [93].

### 4.2. Cobalt in Radiotherapy and Teletherapy

The isotope ^60^Co is useful as a γ-ray source, produced by bombarding ^59^Co with neutrons in a nuclear reactor. ^60^Co has been used in the inspection of industrial materials to reveal internal structure-detecting flaws, destroy cancer cells, and shrink tumors. ^60^Co is used in internal beam radiotherapy, the sterilization of medical equipment, radiation treatment for food, and pest insect sterilization [94]. Another use of ^60^Co is the treatment of different types of cancers using a beam of γ-ray ^60^Co teletherapy machines as a radiation source for medical radiotherapy [95].

### 4.3. Cobalt Nanoparticles

Nanotechnology uses physical and chemical processes to obtain nanomaterials thatå are from 10 to 500 nm in diameter with a high surface-to-volume ratio. Co and Co_3_O_4_ nanoparticles have attracted considerable attention because they are potentially useful for the treatment of microbial infection and for biomedical applications. Co nanoparticles are not toxic in the body at low concentrations and present high antimicrobial activities against bacteria and fungi [96]. The surface of Co nanomaterials interacts with the outer membrane of bacteria, damaging the membrane and inhibiting enzyme activity and bacteria growth [97]. Co nanoparticles do not show cytotoxicity against human cells and do not exhibit deleterious effects in the blood. Omran and coauthors (2020) [98] synthesized cobalt oxide nanoparticles with an average size of 20–30 nm with considerable antimicrobial activity against *Aspergillus brasiliensis* ATCC 16404. Shahzadi and collaborators (2019) [96] studied the effect of Co nanoparticles using *Celosia argentea* plant extract. These Co nanomaterials showed considerable antibacterial activity against *Bacillus subtilis* and *Escherichia coli*. Cobalt oxide nanoparticles, synthesized by Anuradha and Rayi (2019) [99] using *Hibiscus rosa-sinensis*, have been tested against *E. coli*, *Streptococcus mutans*, *Staphylococcus aureus,* and *Klebsiella pneumoniae* with considerable positive results. Co nanoparticles can be used as effective carriers for cytotoxic drugs against tumor cells. 

Ajarem et al. (2022) [100] developed Co_3_O_4_ nanoparticles containing red algae extract, showing anticancer activity against HepG2 hepatocarcinoma cancer cells (IC_50_ of 41.4 μg/mL) and anticoagulant activities, due to inhibition of enzymes that catalyze thrombin formation. Co nanoparticles, prepared using *Euphorbia tirucalli* stem extract by Kgosiemang et al. (2020) [101], showed high cytotoxicity against MCF-7 cells of breast cancer (IC_50_ < 10 μg/mL). Other important uses and applications of Co nanoparticles are in catalysis. An important process catalyzed by Co nanomaterials is the hydrolysis of sodium borohydride (NaBH_4_) to generate hydrogen. Gao et al. (2019) [102] synthesized Co nanoparticles with metallic Co incorporated into carbon materials doped with nitrogen to produce hydrogen by hydrolysis of NaBH_4_. However, this system demonstrated low efficiency, specifically 32.5% after five hydrolysis cycles. The hydrolysis of NaBH_4_ in the presence of Co nanoparticles is also used to produce hydrogen in the process of decomposition of various dyes. Many researchers have described the preparation of Co nanoparticles as catalysts for many reduction cycles in the production of hydrogen. Li et al. (2020) [103] synthesized cobalt oxide (CoO) nanoparticles encapsulated in graphite, with a catalytic efficiency of 82.5% also after 20 cycles of treatment. Wu et al. (2018) [104] studied the decomposition of methylene orange with Co nanoparticles encapsulated in nitrogen-rich carbon nanotubes. After four cycles using this system, the efficiency of reusing this catalyst is still high, with a productivity loss of 96 to 86%. Rasheed and collaborators (2019) [105] studied cobalt oxide nanoparticles obtained from an extract of *Taraxacum officinale* for the catalytic decomposition of methylene orange and direct yellow 142 dyes. This system of nanomaterials catalyzed the reduction of an azo group with an efficiency of 96%. An important result of removing methylene blue and Congo red dyes from wastewater was obtained by El-Sayed et al. (2021) [106] using nanoporous membranes from cellulose acetate, polylactic acid, and biodegradable polyurethane impregnated with Co nanomaterial. This process was carried out under UV radiation. Another application of a Co nanocatalyst is in the Fischer–Tropsch process to convert hydrogen and carbon monoxide into liquid long-chain aliphatic hydrocarbons. 

## 5. Cobalt and Its Toxicological Implications

Inhalation of ambient air and ingestion of food and drinking water containing Co compounds are the main intake routes in humans. The main sources of cobalamin are meat, eggs, clams, oysters, liver, fish, and milk [107]. In humans, the dietary intake of Co varies between 5 and 50 μg/day, most of it in the inorganic form. Vitamin B_12_ represents only a small fraction of oral Co intake. The recommended daily dietary allowance (RDA) of vitamin B_12_ for adults is 2.4 μg/day, which contains 0.1 μg of Co [108]. The permissible concentration of Co in livestock wastewater and irrigation water is 1.0 and 0.05 mg/L, respectively. The allowed limit of Co concentration in drinking water is less than 1–2 mg/L. For this reason, food and beverages consumed by humans offer a potentially active pathway to exposure to toxic and nutritionally needed trace and minor elements. Thus, the monitoring and determination of Co concentration in various food samples such as soft drinks and food is necessary for protecting public health [109]. However, there are concerns about Co being misused as a blood doping agent by athletes to enhance aerobic performance, and some energy drinks may contain high amounts of vitamin B_12_ [110]. 

A recent study by Postnikov et al. (2022) [111] demonstrated that after oral intake of cobalamin at a therapeutic dose significantly exceeding the recommended daily dose, there was a regular slight increase in the blood concentration of total Co (about 1.1 times). At the same time, intake of dietary supplements containing Co in the form of sulfate (CoSO_4_) or asparaginate (about 100 μg per day in terms of pure Co) determined a 4.0–6.7-fold increase in the concentration of total Co, while unchanged vitamin B_12_ plasma concentration was observed. Thus, the monitoring of vitamin B_12_ and total cobalt levels is often requested to detect possible abuse of Co salts for anti-doping control. Several techniques are used for the detection of cobalamin and/or cyanocobalamin in food or pharmaceutical products, including radioscopy, chemiluminescence, high performance liquid chromatography, mass spectrometry, atomic absorption, capillary electrophoresis [112], and recently an electrochemical DNA biosensor and an electro-chemical sensor have been proposed [113,114]. 

The oral bioavailability of inorganic Co compounds varies between 5% and 45% according to their respective water solubility. In occupational settings, workers can be exposed by inhalation to mainly inorganic Co compounds [115]. The lower limit of tolerable daily Co intake set by the Agence Française de Sécurité Sanitaire des Aliments (AFSSA) is 1.6–8 μg/kg bw/day (bw = body weight) [109]. The established Permitted Daily Exposure (PDE) levels for oral exposure recommended by the ICH (ICH guideline Q3D (R1)) for Co is 50 µg/day [116]. Recently, a biokinetic model has been proposed for the characterization of the dose–response relationship and effects of chronic exposure to Co. According to this model, systemic effects are unlikely to occur at Co levels below 300 μg/L (100 μg/L respecting a safety factor of 3) in healthy individuals [110]. Since many Co substances are placed on the market as powders, there is a test requirement under EU law (REACH; EU Regulation (EC) No 1907/2006) to investigate their acute inhalation toxicity [117,118]. 

According to Chen and Lee (2022) [119], the single toxic dose of Co and its salts is not known. In the cohort of patients with “beer drinker’s cardiomyopathy,” it was determined that patients were taking an average of 6 to 8 mg of CoSO_4_ per day for weeks or months. These patients developed severe toxicity, often leading to death. In contrast, infants treated for anemia received 40 mg of CoCl_2_ per day for three months and did not develop toxicity. Urine Co levels are most commonly used for occupational monitoring. Normal serum Co is reported to be 0.1 to 1.2 µg/L. The normal reference range for urine Co is 0.1 to 2.2 µg/L. 

Co exerts toxic effects on the thyroid, heart, and hematopoietic systems and is often considered responsible for occupational lung disease, allergic manifestation, and tumor formation. Carcinogenic manifestation of Co has been described in the International Agency for Tumor Research (IARC) report (IARC 2006) [120], in which Co is classified as group 2A (probably carcinogenic). Acute toxicity is rare, often resulting from excess nutritional supplementation, whereas chronic toxicity can occur due to occupational exposures and from the failure of metal-on-metal hip prostheses [119]. A systematic review about prosthetic hip-associated Co systemic toxicity (PHACT), which is caused by elevated blood Co concentrations after hip arthroplasty, has been recently reported [121]. Recently, cobalt toxicity-related cardiomyopathy has been reported after bilateral metal-on-metal hip arthroplasties for early onset osteoarthritis, which required cardiac transplantation [122]. A case of arthroprosthetic cobaltism resulted in the death of the patient [123]. Other cases of poisoning and systemic cobalt toxicity due to hip implants have been recently described by Świątkowska et al. [124].

CoCl_2_ has been applied in medicine in the treatment of anemia and infections, as well as in sports because it enhances the synthesis of erythropoietin which increases the level of erythrocyte in blood improving sports performance. Today, this therapy is not used on account of adverse effects, like goiter, myxedema, and heart failure. In the past, Co toxicity was related to pulmonary disease in industrial workers who inhaled Co dust, while drilling hard metal or polishing diamonds. 

Nowadays, systemic Co toxicity has been associated with total hip arthroplasty. Co and its alloy with chromium are fundamental components in total hip arthroplasty [125,126] that have been used for more than 40 years. The corrosion of metal ions is the main problem in the use of these implants. In fact, ions released by wear, deterioration, and fretting cause inflammatory and hypersensitivity reactions, bone alteration, and implant failure, and the release of Co and Cr ions into the systemic circulation. Toxicity associated with high levels of metal ions comprises cognitive decline, tinnitus, deafness, atrophy of the optical nerve and retinopathy, cardiomyopathy, hypothyroidism, weakness, and polycythemia [110,127,128]. Co^2+^ in the blood is transported by albumin bound to N-terminal sequence N-Asp-Ala-His-Lys-, similar to Cu and Ni [129]. However, studies of Mothes and Faller (2007) [130] have demonstrated that Co^2+^ also binds to the A and B sites of albumin. The Co binding to albumin is important to study the so-called cobalt–albumin binding test (CAB test). Myocardial ischemia leads to formation of ischemia-modified albumin by cleavage of N-Asp-Ala- (namely, the first two N-aminoacid residues) of the albumin N-terminal. Co, Cu, and Ni bind to ischemia-modified albumin with a lower affinity than native albumin. The CAB test [131,132] consists of adding CoCl_2_ to the patient plasma sample; then dithiothreitol is added, which binds the excess of Co ions not bound to the albumin of the ischemic patients. The test ends with the spectrophotometric determination by titration of the cobalt–dithiothreitol complex compared with a test calibration scale.

## 6. Chelation Therapy for Cobalt Intoxication

There are clinical studies that describe chelation therapy for Co intoxication due to the release of Co ions for the wear of prosthetic hip material. In a pediatric case, an 11-year-old boy, as a consequence of ingestion of several magnets, developed vomiting, weight loss, and a neck mass, and was treated with CaNa_2_EDTA, obtaining a fourfold increase of urinary excretion of Co with clinical improvement [133]. 

Pazzaglia and his collaborators (2011) [134] described a case of a patient with a total hip replacement, who, following the release of Co, Cr, and Mo ions due to wear of the prosthesis, developed optic, acoustic, and peripheral neuropathy from metal intoxication. The patient was treated with EDTA chelation therapy from diagnosis until removal of the implant (74 days) and for a further 33 days. After chelation treatment, the Co level in blood and plasma decreased, while the Co urine levels increased. 

In the Prague General University Hospital, Pelclova and collaborators (2012) [135] described a case of a male patient with ceramic-on-ceramic hip prosthesis. After three years, the implant was replaced by a Co, Cr, and Ti implant. The patient developed neurological, heart, and thyroid toxicity, and hearing loss. He was treated with 2,3-dimercaptopropan-1-sulfonate (DMPS) with increased Co excretion and resolved clinical symptoms. 

Giampreti and his team (2016) [136] followed and described two metal-on-metal hip implant patients treated with N-acetylcysteine to decrease Co and Cr blood levels. This chelating agent uses thiol groups to bind the two metal ions. In both cases, high oral doses of N-acetylcysteine reduced elevated Co and Cr blood concentrations and were well tolerated.

D’Ambrosi and Ursino (2020) [137] treated an asymptomatic patient with high blood levels of Co and Cr with chelation therapy using *N*-acetylcysteine. The levels of Co and Cr ions in the blood, after oral treatment with 1200 mg/die of *N*-acetylcysteine (two tablets of 600 mg every 12 h), were lowered considerably without negative effects.

## 7. Environmentally Friendly Technology for the Reduction of Co Contamination

Phytoremediation, phytoextraction, and rhizofiltration are some techniques used to reduce the amount of Co [138,139,140,141]. The phytoremediation technique uses green plants and their microorganisms to decrease pollution in soils, ground water, surface water, and sludge. Phytofiltration removes the contaminant Co from soil and water bodies through the roots of plants, accumulating the metal ion in the shoot system. More than four hundred plants are reported as hyperaccumulators. For their growth, plants need water, adequate temperatures, and macro- and micronutrients.

Macronutrients are nitrogen, phosphorous, and sulfur, while micronutrients are made up of Co, Fe, Cu, and Mn. The accumulation of heavy metals (Cr, Mn, Fe, Co, Ni, Cu, Zn) in agriculture and water bodies, due to natural activities (weathering from rocks, volcanic activity, and forest fires) as well as anthropogenic activities (mining, cement plants, fertilizers, industrial waste, burning coal to produce energy, and the motor fuel combustion process) is a major concern. The heavy metals are not degradable and their presence in the soil and water can contaminate drinking water, vegetables, and fruits with deleterious effects on human health. 

The plant demand for Co is low, and this metal is absorbed from soil most often as a divalent ion. Co is toxic even at low concentrations because of its non-biodegradable nature, long biological half-life, and capability of accumulating in living systems. Excessive deposit in the soils may result in soil contamination and also lead to uptake by crop plants, affecting the quality of plants, vegetables, and fruits, and the safety of human beings. Co at low concentrations plays essential roles in the growth of plants and the development of root nodules, but at high levels, causes toxicity to the plants [142]. High concentrations of this metal inhibited the activities of catalase and peroxidase (antioxidant enzymes), ribonuclease, and acid phosphatase in tomato plants [143]. The toxicity in plants depends upon plant species, type, and the pH of the soil, and form in which Co is present in the soil. 

The phytoremediation process is very popular because it is a low-cost and friendly technology for remediating contaminated environments (soil, groundwater, and surface water). High Co concentrations in the soil have toxic effects like leaf fall, inhibition of greening, discolored veins, and reduced shoot weight. Furthermore, excess Co has detrimental effects on plant metabolic functions, leaf necrosis, interveinal chlorosis, inhibition of cellular mitosis, and seed generation [144]. Co concentration in plants is normally 0.05–5.0 mg/kg dry weight, but this value grows considerably (120–240 mg/kg dry weight) in plants close to mining areas. To counteract Co toxicity, plants have developed several mechanisms to detoxify or accumulate this metal, among which are various bioremediation strategies. 

The study of Malik et al. (2000) [145] reported that *Alyssum murale* and *Alyssum corsicum* demonstrated excellent results in the phytoextraction technique. The maximum Co quantity extracted by *Alyssum murale* is 1320 mg/kg dry weight, while the Co quantity accumulated by *Alyssum corsicum* is 1080 mg/kg dry weight. *Alyssa selvatica* showed remarkable Co accumulation in the leaves (800 mg/kg dry weight). The experimental results of Prajapati et al. (2012) [146] showed that the water lettuce *Pistia stratiotes* L. is capable of efficiently removing Co and Cr from water. 

Lotfy and Mostafa (2014) [147] studied the use of five different plant species to extract Co and Cr out of two heavily polluted soils north of Cairo (Egypt). Five plant species, namely, *Panicum antidotal*, *Pennisetum purpureum*, *Cucurbita pepo*, *Gossypium hirsutum,* and *Helianthus annuus*, were grown on Mostorund clayey soil (irrigated with contaminated water for more than 30 years) and sandy polluted soil from the El-Gabal El-Asfar (subjected to sewage water irrigation for more than 50 years). After 60 days, plant roots exhibited higher Co and Cr accumulation than shoots, by 1.32–2.25 and 1.7–2.34 fold, respectively. *H. annuus* roots showed higher Co and Cr accumulation, followed by *P. antidotal*, *P. purpureum*, *G. hirsutum,* and *C. pepo*. 

Van der Ent and collaborators (2018) [148] discovered that *Glochidion* cf *sericeum* from Sabah (Malaysia) is capable of simultaneously accumulating Ni and Co (1500 mg/kg) in the leaves. The chemical speciation of these two metals is associated with citrate for Ni and tartrate or malate for Co.

The team of Khalid (2020) [149] studied the physiological and biochemical responses along with the phytoextraction capability of *Althernanthera bettzickiana* for contaminated soil in the presence of copper and Co. Healthy plants of *A. bettzickiana* were placed in pots with 5 kg of sandy loam soil. Plants were treated with 2.5, 5.0, 7.5, and 10.0 mM Cu and Co. After 8 weeks of treatments at these different concentrations of both metals, plant growth, chlorophyll content, and antioxidant enzyme activities were measured without signs of toxicity compared to the control for both metals. Co and copper concentrations and accumulation increased with increasing levels of both metals in plant shoots, stems, and leaves compared to the control up to 7.5 mM and then decreased slightly at 10.0 mM. 

## 8. Conclusions

Co, along with iron and nickel, is a ferromagnetic metal, belonging to Group 9 (VIII) of the periodic table of elements. Co is an essential trace element of fundamental importance for human health due to its role in vitamin B_12_. It is also present in at least eight noncorrin Co-dependent enzymes. It is classified as group 2A (probably carcinogenic) in the IARC report (2006). Co is present in some foods and drinking water. Moreover, Co-containing alloys are utilized for hip and knee replacement and for dental prosthesis. ^60^Co, a γ-rays source, is used for the sterilization of medical equipment, for food and pest insect sterilizations, and for medical radiotherapy. Co nanoparticles are used in a range of treatments of microbial infections and biomedical and biological applications, including their use as carriers for cytotoxic drugs against tumors, since they do not exhibit harmful effects in the blood. A low intake of vitamin B_12_ can cause different health problems, such as fatigue, anemia, kidney failure, liver disease, or neurotoxicity. On the other end, high Co concentration poses a risk to eyes, thyroid damage, heart disease, decreased pulmonary function, tinnitus, deafness, cardiomyopathy, and hypothyroidism. This review highlights the uses and major routes of exposure to Co, as well as certain molecular mechanisms related to its toxicity. A focus on cobalamin, the vitamin containing Co, has been emphasized, along with the outcomes deriving from its deficiency and/or excess in humans. Furthermore, chelation therapy as well as phytoremediation methods for the removal of Co from soils are described. Finally, monitoring environmental exposures and hazards to Co should be performed to counteract its toxicity, which requires attention for human health.

## Data Availability

Not applicable.
